# Antibiotic Spacer Arthroplasty for Revision MTP Arthrodesis: A Novel Means to Build the Implant: A Case Report

**DOI:** 10.7759/cureus.537

**Published:** 2016-03-21

**Authors:** Adam Bitterman, Cristin Mathew, Milap Patel, James P Gurtowski

**Affiliations:** 1 Orthopaedic Surgery, Rush University Medical Center; 2 Orthopaedics, North Shore Long Island Jewish Health Systems

**Keywords:** hallux rigidus, metatarsophalangeal osteoarthritis, mtp, metatarsophalangeal arthrodesis, metatarsophalangeal arthroplasty, metatarsophalangeal arthrodesis failure, antibiotic spacer

## Abstract

Metatarsophalangeal (MTP) joint osteoarthritis (OA), also known as hallux rigidus (HR), is the most common degenerative arthropathy of the foot and is often the result of trauma. There are multiple methods of addressing the patient’s pain and limited function. Arthrodesis is the gold standard to manage severe MTP arthritis with a highly significant union rate. With various techniques of arthrodesis available, ranging from cannulated screw fixation, Kirschner wires, as well as plate and screw fixation, the orthopedic surgeon has multiple modalities to address this ailment; however, when these fail due to infection, the armament is limited. Through the idea of articulating antibiotic spacers in other regions of the body such as the knee and hip, we present a novel technique to the creation of an antibiotic spacer in the setting of a failed infected MTP arthrodesis.

## Introduction

Metatarsophalangeal (MTP) joint osteoarthritis (OA) affects 2.5% of patients older than the age of 50. First described in 1887 by Davis-Colley, it was later named hallux rigidus (HR) by J.M. Cotterill [1]. In patients with unilateral presentation, the etiology is often sequelae of a traumatic event. Marked with pain and limited sagittal motion of the joint, patients with HR often look for treatment to include increased function and ultimately a better quality of life. Non-surgical methods of treatment include anti-inflammatory medicine, whether oral or injectable, shoe modifications, carbon fiber inserts, and use of rocker bottom soles. After conservative measures have failed, often surgical intervention is required, ranging from joint-sparing procedures such as cheilectomy and periarticular osteotomies, to joint-altering excisional procedures including Keller resection arthroplasty, interposition arthroplasty, total joint arthroplasty and hemiarthroplasty, to joint-destruction procedures like arthrodesis [2].

Arthrodesis, being the standard of care for severe hallux rigidus, can be accomplished through various methods. These techniques include fixation with Kirshner wires, staples, as well as plates and screws. Although successful, arthrodesis has proven to have a significant complication rate. Complication rates have ranged from 0% to 8.9% [3-7]. In a study of 34 patients with Regnauld class II hallux rigidus, Beertema et al. found an 8.8% nonunion and 11.7% revision rate [8]. A failure secondary to infection can be devastating for the patient and the surgeon, often necessitating removal of hardware for eradication of the infection. Unfortunately this leaves the joint painful, unstable, and nonfunctional. We present a novel technique for the creation of a customizable antibiotic spacer in this tragic setting that provides patients a stable and functional MTP Joint. 

## Case presentation

**Figure 1:**
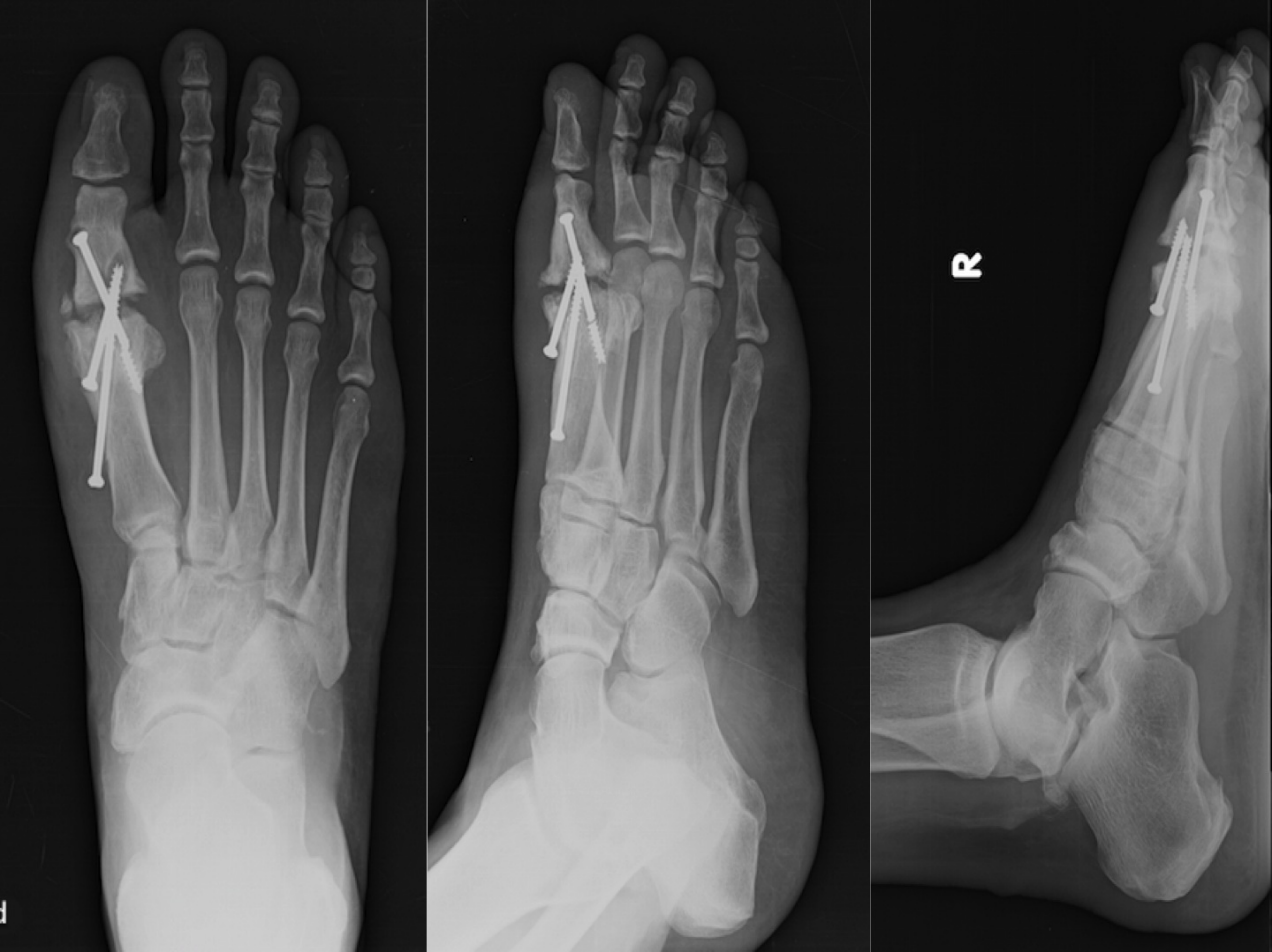
Radiographs one year post arthrodesis AP, oblique, and lateral radiographs of the patient's right foot status post MTP arthrodesis with lucencies surrounding the cannulated screws as well as a broken screw demonstrating a non-union of the right first MTP joint.

We present a case study of a 56-year-old community ambulating male who endured a failed MTP arthrodesis due to infection, whom we treated with a novel method of creating an articulating antibiotic impregnated cement spacer. The patient had undergone a right first MTP arthrodesis with fixation via three 4.0 mm cannulated screws by another provider one year and four months earlier. A month following the arthrodesis, the patient was found to have a purulent draining wound with cultures growing Methicillin-Resistant Staphylococccus Aureus (MRSA). During the course of the year after diagnosis, the patient was placed on suppressive antibiotics and he managed his wounds with appropriate means for the intermittent drainage. Postoperative radiographs after a year demonstrated a lack of definitive fusion and lucencies surrounding the screw implants indicative of osteomyelitis as seen in Figure 1.

**Figure 2:**
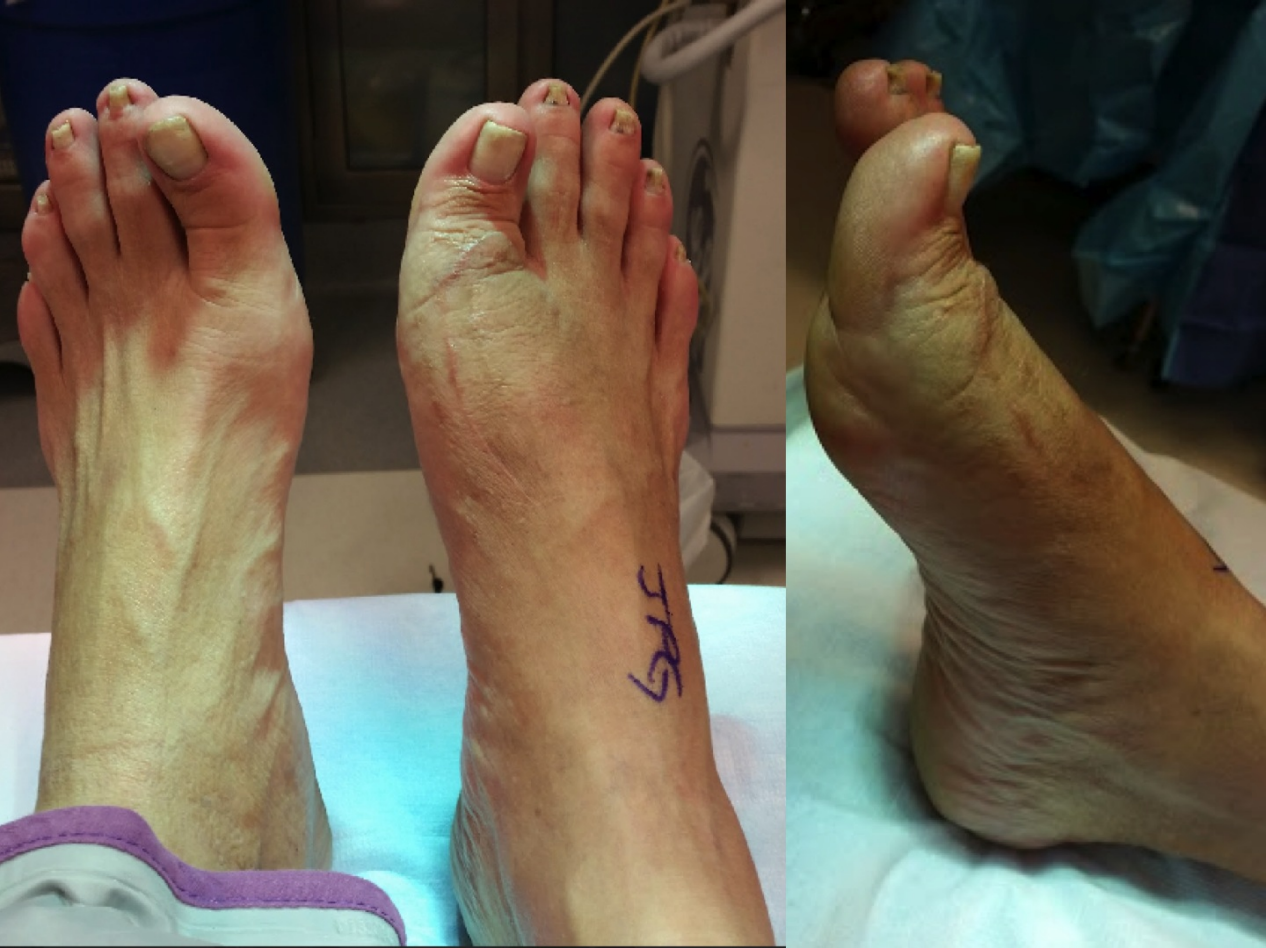
Preoperative holding presentation Demonstrating physical presentation of bilateral feet prior to surgery. Note the shortening, swelling, and erythema of the right hallux.

On physical exam, his right first ray was swollen, erythematous, and warm without any open wound or sinus tracts. The patient was neurovascularly intact and an initial radiograph in the office revealed failed hardware. Due to his history and clinical presentation as well as the concern for potential seeding to his hip arthroplasty implants, recommendations to remove the foot hardware and eradicate the infection with placement of an antibiotic spacer were presented. After a thorough discussion of alternative measures, and risk and benefits, the patient agreed to move forward with the removal of hardware and placement of an antibiotic spacer. A preoperative presentation of the patient's foot is seen in Figure 2.

The patient was aware and agreeable to the case presentation, and consented during the time of the operative procedure.

### Operative technique          

**Figure 3:**
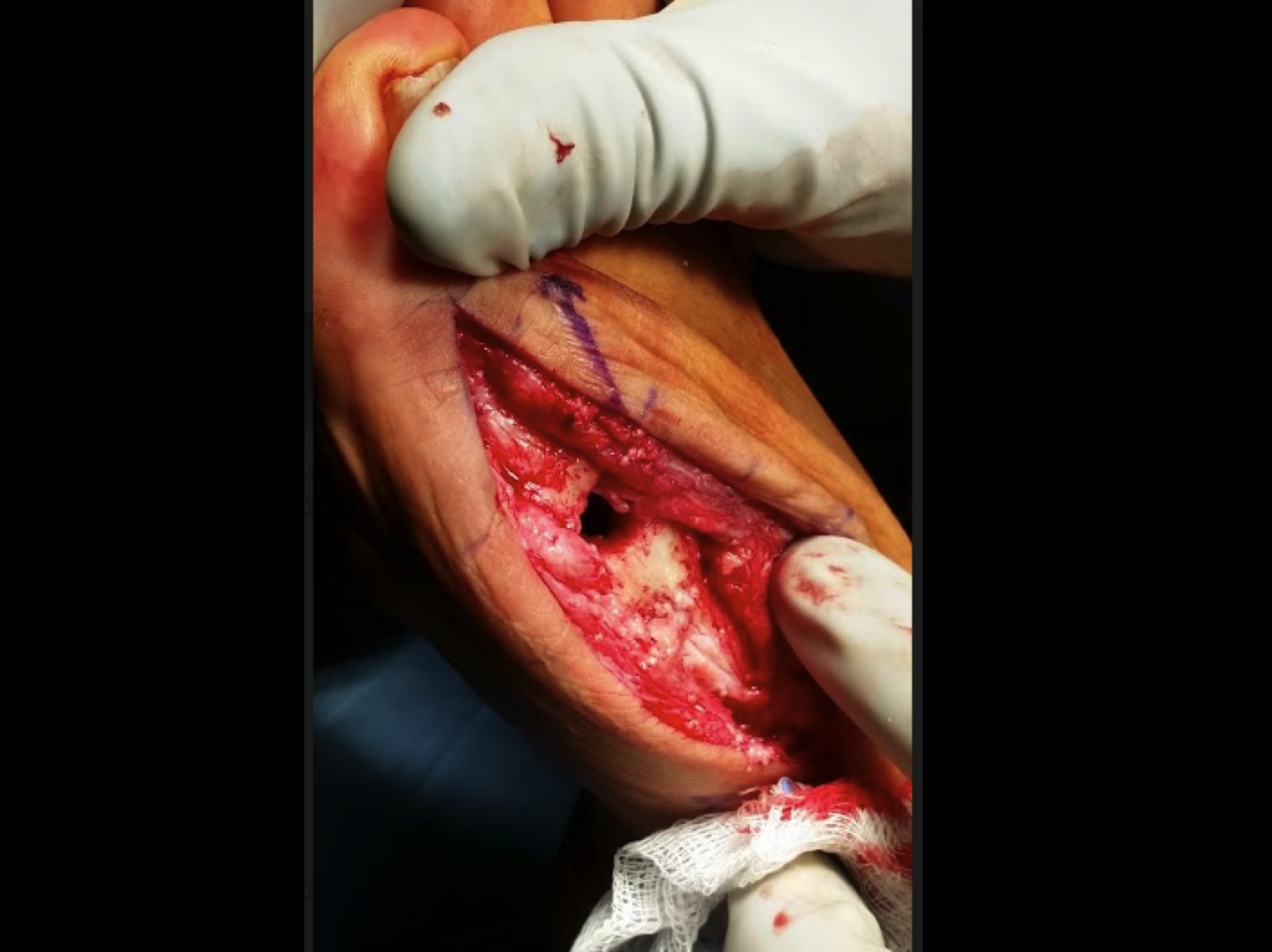
Implant removal and bone resection Intraoperative photograph demonstrating proximal and distal resection of the MTP joint.

A dorsal midline incision was made incorporating the previous incision, and the first MTP capsule was then incised allowing visualization of the area of nonunion. After identifying the osteomyelitis around the hardware and the metatarsophalangeal joint, the two intact screws were removed under fluoroscopic guidance. The final screw fragments were removed with the trephine technique by flexing the interphalangeal (IP) joint and creating a core to pull out the proximal piece. Gross purulence was noted medially over the broken hardware, which was sent for culture and speciation along with the removed hardware.The MTP joint was then resected proximally and distally to remove all portions of diseased bone as seen in Figure 3.

**Figure 4:**
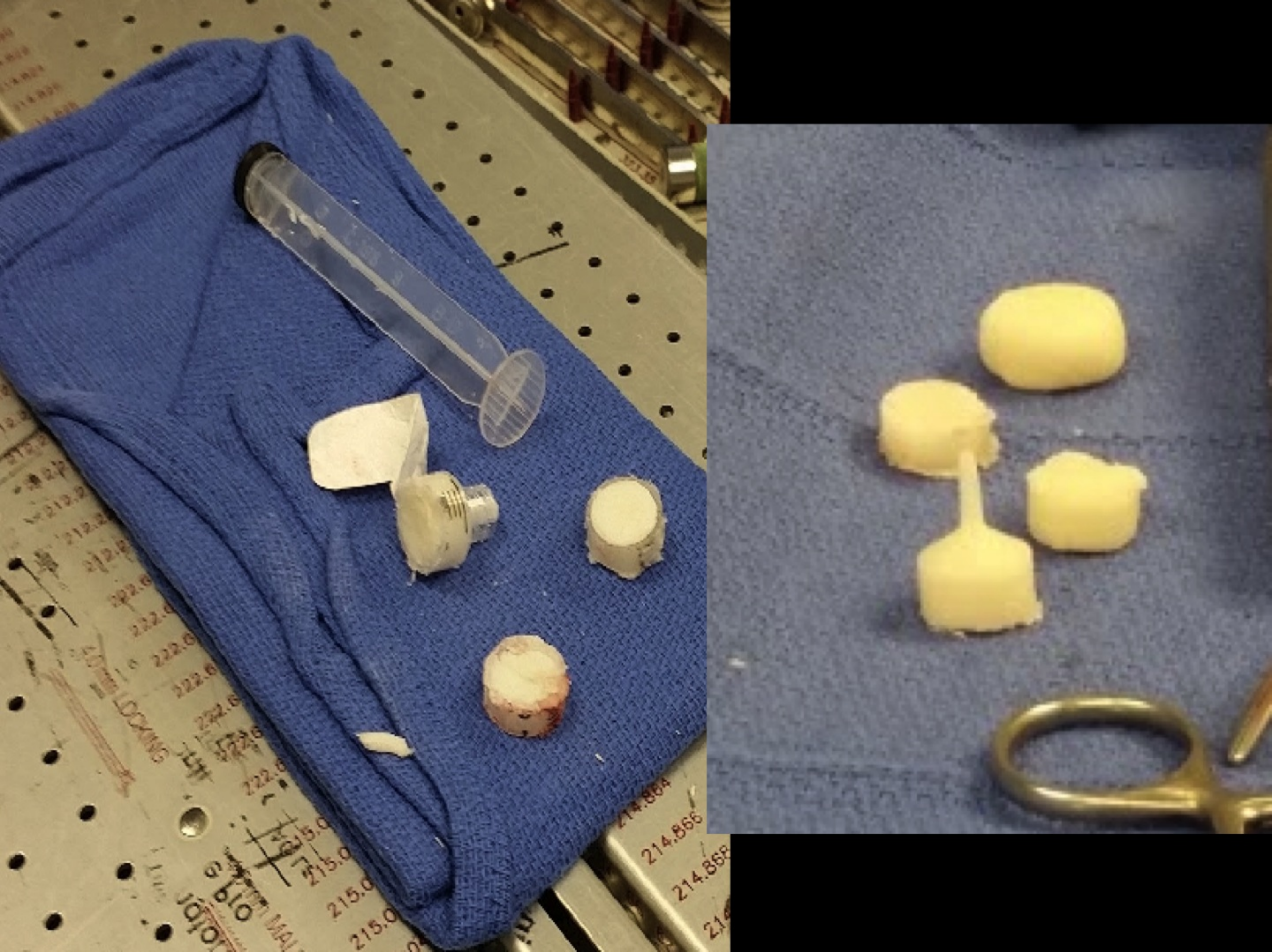
Creation of customizable cement spacer Intraoperative photograph demonstrating the cement spacer created from a 10 cc syringe after it was cut with a sagittal saw. Note the keel of the spacer created with the cement compacted into the distal tip of the syringe. Intraoperative photograph demonstrating the keeled antibiotic spacer and articulating spacers varying in size.

**Figure 5:**
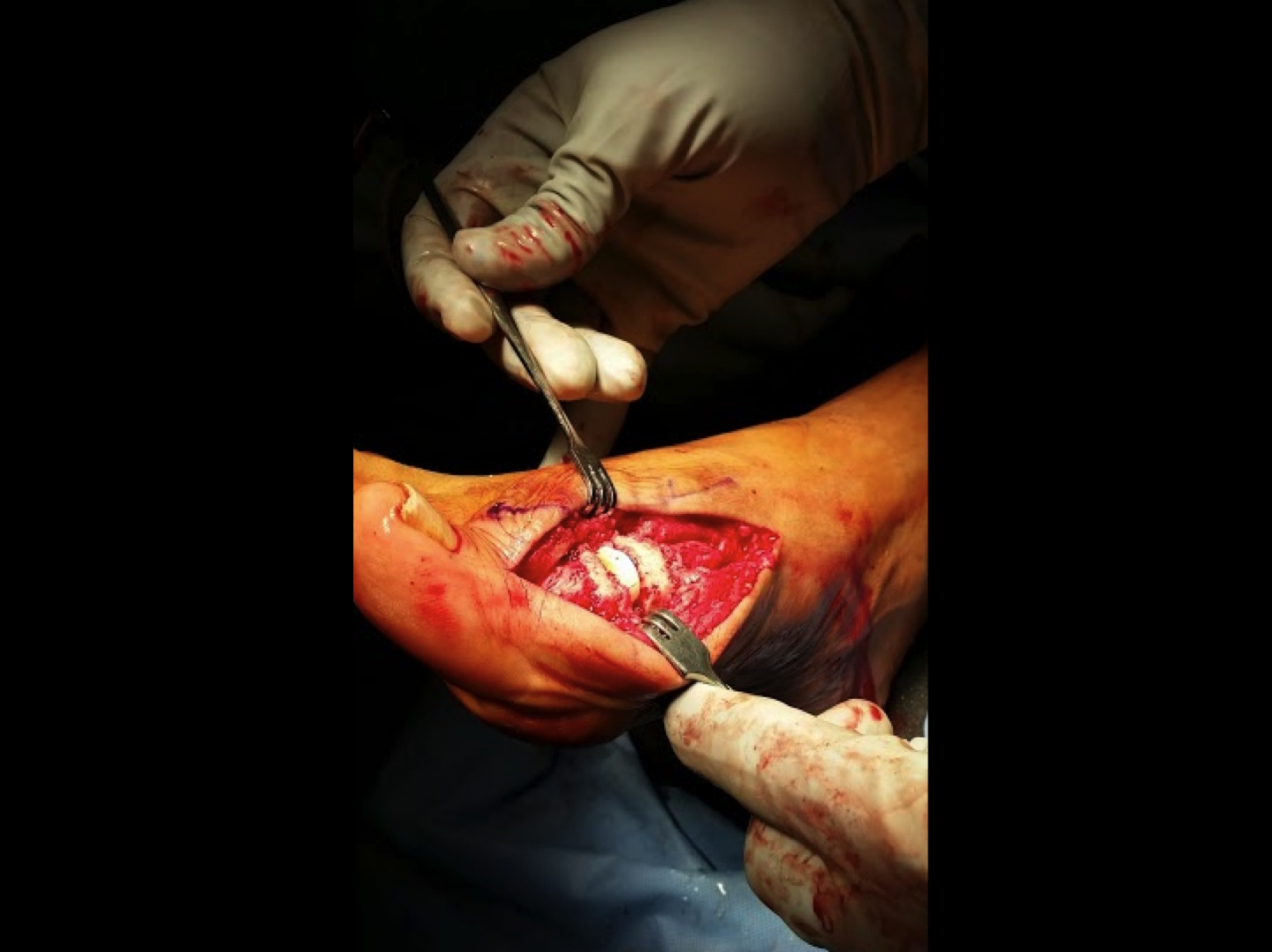
Spacer implantation Photograph demonstrating intraoperative placement of the antibiotic spacer in the MTP joint.

An antibiotic spacer was created using 1 gram of vancomycin, 40 grams of bone cement, and a 10 ml syringe for molding purposes. The antibiotic impregnated cement was inserted into the syringe. Using the plunger within the syringe, a concave surface was created by the abutting rubber portion of the plunger. An antibiotic spacer stem/keel was fashioned through the cement that was pushed into the tip of the syringe. Once the cement had hardened, the plastic of the syringe including the narrow tip was removed using a micro-sagittal saw, providing an 8 mm cylinder of cement. The mold was resected to be approximately 8 mm in length to maintain the length and orientation of the joint, and the narrow tip provided a keel for insertion into the metatarsal as seen in Figure 4. The MTP joint was then thoroughly irrigated with 3 liters of antibiotic impregnated solution, and the spacer was then inserted (Figure 5).

**Figure 6:**
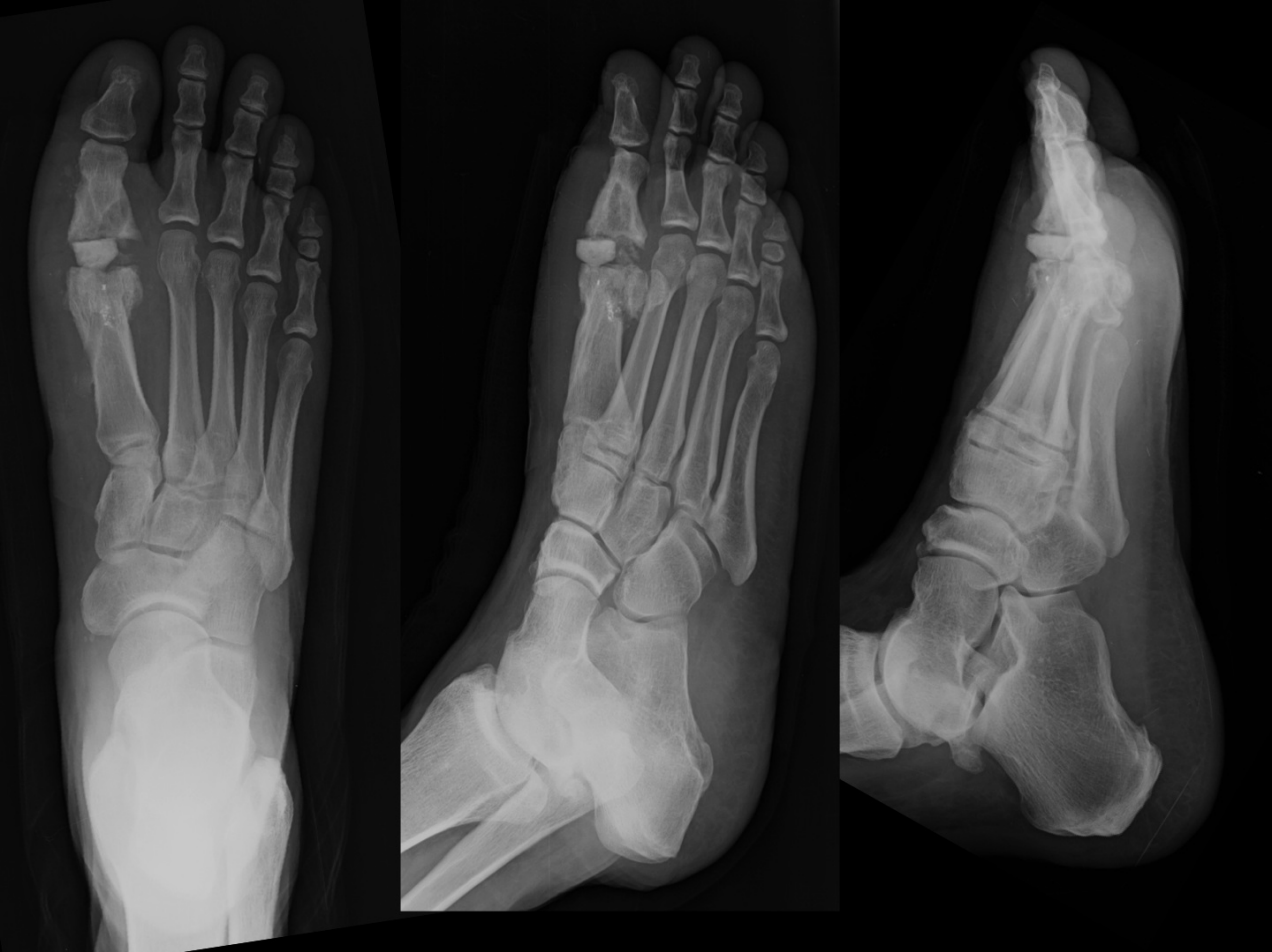
Postoperative radiographs Postoperative AP, oblique, and lateral foot radiograph demonstrating the placement of the articulating antibiotic spacer within the first MTP joint.

The joint was assessed for proper tensioning and stability through limited ranges of motion and stressing in all planes. The capsule was closed with 3-0 vicryl suture, and the skin was closed with 3-0 nylon suture. Xeroform was placed over the incision and a dry sterile dressing was applied with an overlying posterior mold with sugartong fiberglass splint. While in recovery, post operative radiographs were taken (Figure 6).

Postoperatively, the infectious disease team saw the patient and a peripherally inserted central catheter (PICC) line was placed for administration of intravenous vancomycin and ceftriaxone. On postoperative day number 1 a baseline erythrocyte sedimentation rate (ESR) was collected which measured 34 mm/hr (Normal 0-28 mm/hr) and serum white blood cell count (WBC) of 8,800/uL (Normal 4,500-10,000/ uL). Intraoperative cultures remained negative for bacteria and fungi. His clinical stay remained uneventful and he was discharged home on postoperative day number 4.

He presented to the office for follow-up one week postoperatively with a clean and dry wound without drainage or evidence of cellulitis. The PICC line was functioning well and the patient was placed in a hard soled shoe with eventual progression to a walking boot. On postoperative day 15, no signs of infection were noted and his sutures were removed after successful wound healing.

One month postoperatively, the patient presented to the ED with complaints of his PICC line bleeding. While being evaluated in the ED, the patient was found to have ipsilateral thromboses within the axillary and subclavian veins. The patient was placed on a blood thinning regimen, which included lovenox and a coumadin bridge. The patient was subsequently changed from intravenous ceftriaxone and vancomycin to oral doxycycline. The thrombosis was attributed to the underarm trauma from the use of crutches and the patient was provided education and physical therapy with the use of a walker. At his most recent follow-up, approximately one year postoperative, he denied any pain to his foot and was adamantly against having the cement implant removed. He continues to have an active lifestyle, which is free of any limitation.

## Discussion

MTP arthrodesis is typically used as a salvage procedure in the cases of failed cheilectomy, resection arthroplasty or implant arthroplasty. It is also considered the gold standard treatment for end-stage arthritis of the MTP joint or hallux rigidus as in the case for our patient. However, very little evidence for the management of failed arthrodesis exists in the presentation of a failed infected arthrodesis.

Revision surgeries for hallux deformities are often complex and challenging. A salvage procedure must address a solution for all elements of deformity. Arthrodesis of the first MTP joint is a salvage procedure that provides this. Fusion has been known to consistently provide significant pain relief and restoration of function for treatment of arthritis with a union rate approaching greater than 90% [9-11]. Questions remain regarding the treatment options when an arthrodesis of the MTP joint fails.

There is very limited published data regarding treatment options for failed MTP arthrodesis; even more limited when an arthrodesis fails secondary to infection. When the complication of infection is added, placement of hardware in a septic joint or within osteomyelitis is not recommended. 

Our patient’s presentation and desires for postoperative function without amputation brought out some unique issues regarding his management. With a chronic infection within the joint and bone, treating the infection was our first priority. This would entail removal of all the hardware and debridement of nonviable bone. Secondly, with a relatively active lifestyle, the patient desired to achieve more function without sacrificing the joint’s range of motion, leading to arthroplasty over arthrodesis. Unfortunately, infection and the patient’s poor bone stock significantly hindered the placement of new hardware. 

Our development of an articulating antibiotic spacer helps provide local antibiotics to eradicate the infection while providing an inexpensive modular implant to maintain metatarsal length. Through the use of an articulating spacer, the patient is able to maintain joint length and stability, thus decreasing the risk of transfer metatarsalgia. Once the infection is eradicated via the antibiotic spacer and intravenous antibiotics, if the patient continues to have pain and decides to have a revision, the antibiotic spacer can be removed and a revision arthrodesis can later be completed.

Management strategies of revision arthrodesis often result in significant debridement and bone loss. As a result of the altered anatomy, the weight-bearing surface of the foot is changed, which may result in metatarsalgia. Another common complication of MTP arthroplasty is hardware subsidence, which would be a higher risk in the case of osteomyelitis and poor bone stock. As is done when faced with a similar situation in other joints of the body, we looked at the use of articulating antibiotic spacers of the knee. This treatment option maintains soft tissue length and joint function while also helping to eradicate an infection. 

Designing an articulating antibiotic spacer that would replace the resected portions of infected bone entailed the recreation of the joint surface and adjustment of proper length to maintain preoperative metatarsal length, soft tissue tension as well as proximal and distal fixation. After insertion of antibiotic impregnated cement into a 10 ml syringe, the plunger is used to create a concave surface for the spacer. The very tip of syringe also provides a cement post to allow insertion into the bone and improve fixation. The length of the spacer can be customized based on the amount of bone and joint space that must be reconstructed.

## Conclusions

Failure of MTP Arthrodesis due to infection can be a catastrophic complication with limited options for treatment. Just as treatments in other joints when infections surround implants, the removal of hardware and use of local and systemic antiobiotics are necessary for effective eradication. Unfortunately, with removal of hardware after failed arthrodesis, loss of bone stock can also be a significant sequlae causing altered mechanics of the foot which can lead to significant morbidity. The novel technique of creating a customizable antibiotic spacer with a syringe provides a needed solution to a difficult problem. 
